# Side-by-Side Profiling of Oxazolidinones to Estimate the Therapeutic Window against Mycobacterial Infections

**DOI:** 10.1128/aac.01655-22

**Published:** 2023-03-15

**Authors:** Dereje A. Negatu, Wassihun Wedajo Aragaw, Julianna Cangialosi, Véronique Dartois, Thomas Dick

**Affiliations:** a Center for Discovery and Innovation, Hackensack Meridian Health, Nutley, New Jersey, USA; b Center for Innovative Drug Development and Therapeutic Trials for Africa (CDT-Africa), Addis Ababa University, Addis Ababa, Ethiopia; c Department of Medical Sciences, Hackensack Meridian School of Medicine, Nutley, New Jersey, USA; d Department of Microbiology and Immunology, Georgetown University, Washington, DC, USA

**Keywords:** oxazolidinone, mitochondrial toxicity, tuberculosis, nontuberculous mycobacteria

## Abstract

New oxazolidinones are in clinical development for the treatment of tuberculosis and nontuberculous mycobacterial (NTM) infections, as a replacement for linezolid and tedizolid, which cause mitochondrial toxicity after prolonged treatment. Here, we carried out side-by-side measurements of mitochondrial protein synthesis inhibition and activity against clinically relevant mycobacterial pathogens of approved and novel oxazolidinones. We found a large range of selectivity indices suggesting TBI-223 and sutezolid as promising candidates against tuberculosis and NTM lung disease caused by Mycobacterium kansasii.

## TEXT

Linezolid is an oxazolidinone included in the WHO-recommended regimens to treat multidrug-resistant tuberculosis (MDR-TB) (1, 2). It is occasionally included in the treatment of nontuberculous mycobacterial pulmonary disease (NTM-PD) (3), with tedizolid as a potential alternative (4, 5). Both antibiotics were developed to cure bacterial infections that typically require short-term therapy for 10 to 14 days, and up to 28 days (6). In contrast, successful treatment of MDR-TB and NTM-PD takes a minimum of 6 months and up to several years. Linezolid is efficacious in TB patients with highly drug-resistant disease (7, 8), but prolonged linezolid administration beyond a month leads to peripheral neuropathy and myelosuppression in a large fraction of the patients (2), as the major recognized adverse events, leading to temporary or permanent treatment discontinuation (9). In the highly successful NIX-TB trial (10), efficacy of the bedaquiline-pretomanid-linezolid regimen came at a cost: peripheral neuropathy and myelosuppression developed in 81% and 48% of the patients, respectively. In the follow-up ZeNix trial, a reduction of the linezolid daily dose from 1,200 mg to 600 mg improved the tolerability profile but still resulted in serious adverse events and linezolid dose modification in 24% and 13% of the subjects, respectively (11). Thus, there is a strong incentive to replace linezolid with a safer oxazolidinone for the treatment of mycobacterial lung disease.

Oxazolidinones inhibit bacterial protein synthesis by targeting the peptidyl transferase activity of the 23S rRNA in the 50S ribosomal subunit (12). Mammalian mitochondrial ribosomal components are closely related to their bacterial counterparts, and decreased mitochondrial function correlates with toxicity in the clinic, leading to drug discontinuation (13). Although a correlation between mitochondrial protein synthesis (MPS) inhibition and the *in vivo* toxicity profiles of oxazolidinones has not been firmly established, this assay is broadly used as an accepted surrogate for clinical toxicity (14). Several novel oxazolidinones are in clinical development (Table 1), with the objective of alleviating toxic effects and improving the therapeutic window, either by increasing potency while maintaining MPS inhibition, or decreasing MPS inhibition while maintaining potency, or both. However, a quantitative side-by-side comparison, using standardized protocols, of the MPS inhibition versus potency of oxazolidinones in clinical use or clinical development is lacking. MPS inhibition has been reported for a subset of clinical oxazolidinones and, in most cases, was measured using a different assay, occasionally with linezolid included as a control (Table 1). To address this gap, we assembled a list of commercially available oxazolidinones that are either approved or in clinical development (Table 1) and measured for each the MPS inhibition and potency against major mycobacterial pathogens (Mycobacterium tuberculosis, Mycobacterium kansasii, the Mycobacterium avium complex [MAC], and the Mycobacterium abscessus complex [MAbC]), to generate directly comparable ratios. The data set also provides standardized MPS inhibition values for correlation with appropriate adverse event trial data and evaluation of the MPS assay as a predictive marker of clinical toxicity.

**TABLE 1:** Oxazolidinones in clinical use or at various stages of clinical development

Drug name	Drug abbreviation	Structure	Clinical development stage	Reported MPS inhibition	
				MPS IC_50_ (μg/mL)	Assay and reference
Linezolid	LZD	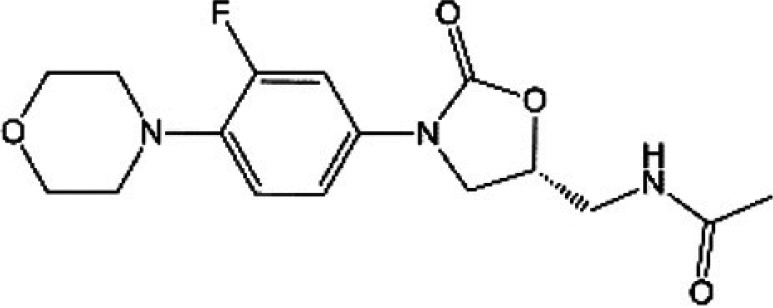	FDA approved for treatment of bacterial infection. Clinically used to treat MDR-TB and NTMs (15–17)	3	Assay not described (18)
				5.4	COX-1/SDH-A formation reduction by ELISA assay (MitoBiogenesis in-cell kit) (19)
				2.2 to 4.3	Protein synthesis in isolated rat heart mitochondria (14, 20)
Tedizolid	TZD	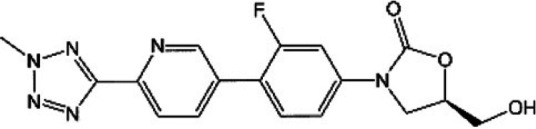	FDA approved for treatment of acute bacterial skin and soft tissue infections (21, 22)	6-fold lower than that for LZD	CYTox1 protein synthesis in HL-60 promyelocytes (23)
				0.12	Protein synthesis in isolated rat heart mitochondria (20)
Sutezolid	SZD	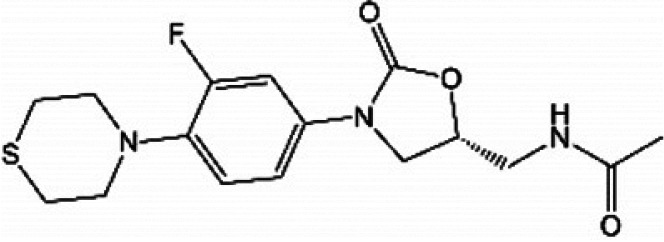	Phase II for treatment of pulmonary tuberculosis (NCT03959566) (24, 25)	4.2, 2.9, 6.6	Protein synthesis in isolated rat heart, rabbit heart, and rabbit bone marrow mitochondria (14)
Sutezolid-M1	SZD-M1	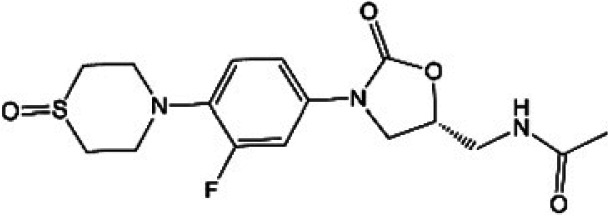	Active metabolite of sutezolid (24)	NR^*a*^	
Delpazolid	DZD	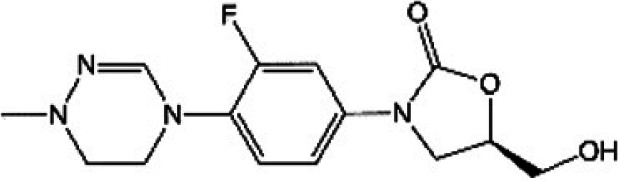	Phase II for treatment of pulmonary tuberculosis (NCT04550832) (26, 27)	1.0 to 3.4^*b*^	Protein synthesis in mitochondria from human leukemia cell line and cardiomyocytes (26)
TBI-223		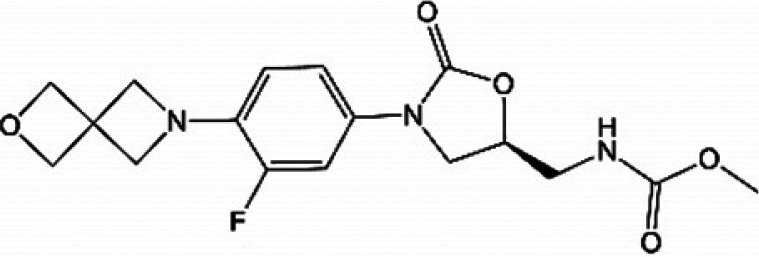	Phase I for treatment of pulmonary tuberculosis (NCT04865536) (28)	>27	COX-1/SDH-A formation reduction by ELISA assay (MitoBiogenesis in-cell kit) (41)
Radezolid	RZD	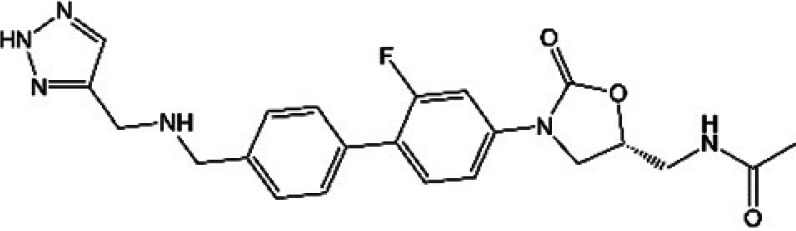	Phase II for treatment of community-acquired pneumonia (NCT00640926)—last updated in 2016 (29)	NR	
Contezolid	MRX-1	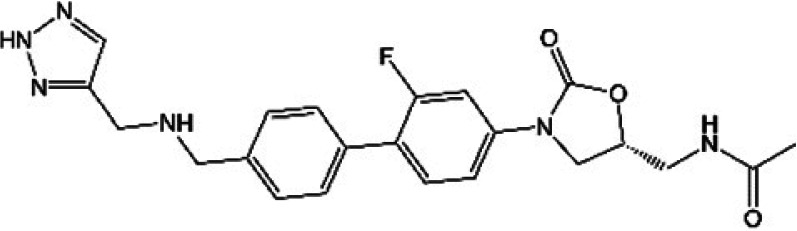	Phase III for diabetic foot infections (NCT05369052). Approved in China for treatment of drug-resistant bacteria (30, 31)	15.7^*c*^	Myelosuppression IC_50_ in human CD34^+^ bone marrow cells (32)

a NR, not reported or not publicly available to our knowledge.

b Same as that for LZD.

c Myelosuppression IC_50_ (not the MPS IC_50_). The IC_50_ of linezolid in the same myelosuppression assay was 7.9 μg/mL.

**FIG 1:**
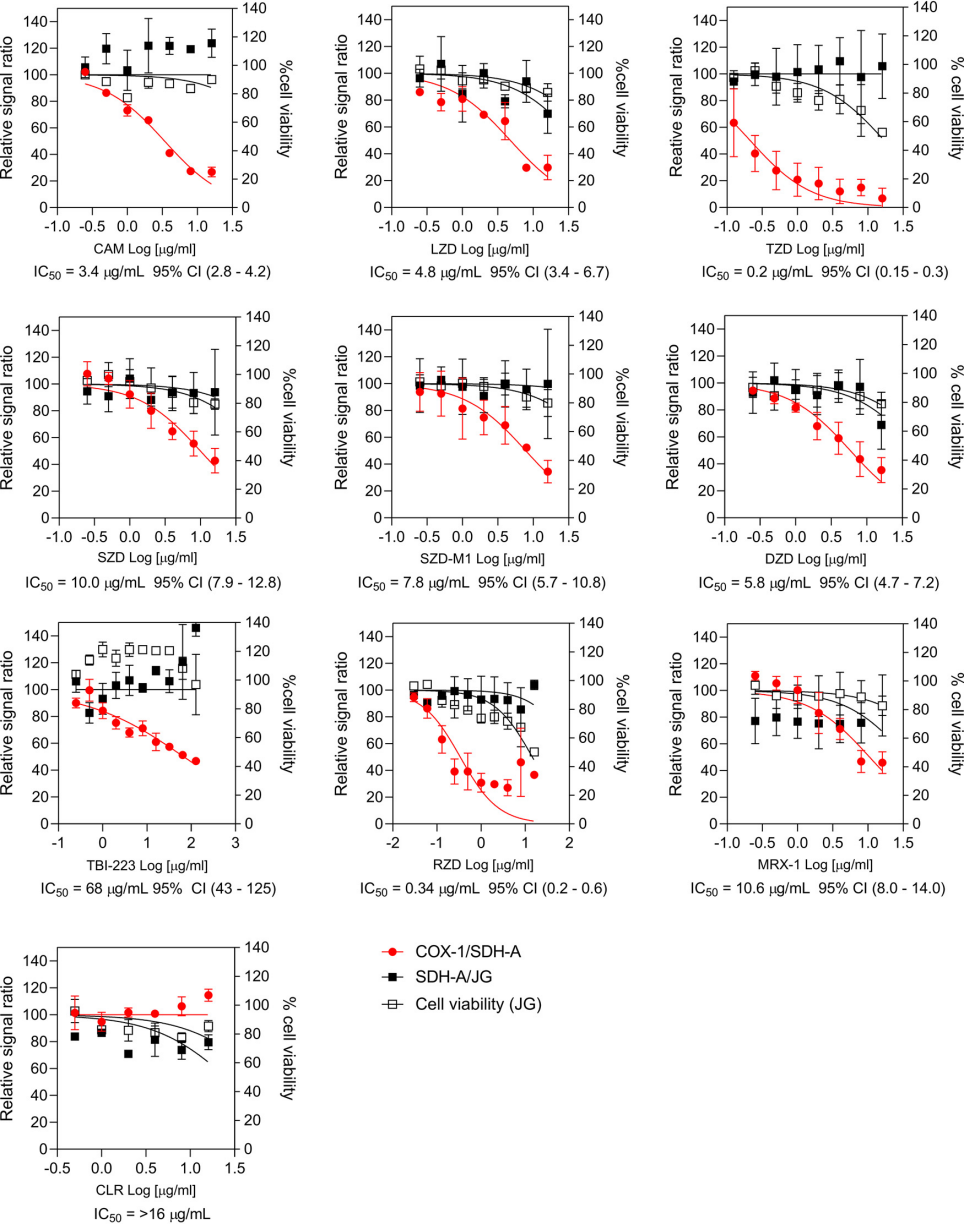
Dose-response inhibition of mitochondrial protein synthesis of clinical oxazolidinones. MPS inhibition was quantified using the MitoBiogenesis in-cell ELISA kit (ab110217; Abcam) in HepG2 cells (see Fig. S1 in the supplemental material). The assay compared the production of two proteins: COX-1, synthesized by mitochondrial ribosomes, and SDHA, synthesized by cytoplasmic ribosomes. Cell viability was measured with the Janus green (JG) stain. Raw data were interpreted per the manufacturer’s instructions and plotted using GraphPad Prism to calculate the IC_50_ values of the relative inhibition of COX-1/SDH-A synthesis, and the percent cell viability. Chloramphenicol (CAM) and clarithromycin (CLR) were used as the positive and negative controls, respectively. The oxazolidinone abbreviations are as provided in Table 1. 95% CI, 95% confidence interval.

To generate MPS inhibition values, we used the mitochondrial biogenesis inhibition assay (MitoBiogenesis in-cell kit; Abcam) and measured the effect of increasing drug concentrations on the synthesis of the mitochondrial DNA-encoded COX-1 and nuclear DNA-encoded SDH-A proteins, detected by enzyme-linked immunosorbent assay (ELISA) (see Fig. S1 in the supplemental material). The COX-1 and SDH-A levels were normalized to viable cell numbers quantified using the Janus green staining method according to the manufacturer’s protocol. The relative COX-1/SDH-A signal ratio was calculated and plotted to infer the 50% inhibitory concentrations (IC_50_) (Fig. 1). Cell viability was determined by Janus green staining of the drug-treated wells relative to the drug-free wells. Oxazolidinones were purchased from MedChemExpress LLC (USA), except for sutezolid, which was provided by Sequella, Inc. (as the active pharmaceutical ingredient used in the recently completed phase IIb trial), and its active metabolite, sutezolid-M1, which was obtained from the NIH reagent program. Chloramphenicol and clarithromycin (Sigma-Aldrich) were used as the positive and negative controls, respectively, and the expected potency patterns were observed in both cases (a chloramphenicol MPS IC_50_ of 3.2 ± 0.2 μg/mL was reported in reference 14). The MPS IC_50_ values of the oxazolidinones ranged from 0.2 μg/mL for tedizolid to 68 μg/mL for TBI-223. Qualitatively, the observed MPS IC_50_ ranking was comparable to published values across the series (Fig. 1 and Table 1).

Next, we measured the potency (MIC) of the oxazolidinones against the etiologic agents of the major mycobacterial lung diseases: M. tuberculosis, M. kansasii, the M. avium complex (MAC), and the M. abscessus complex (MAbC). Staphylococcus aureus was included as a reference organism, since oxazolidinones were originally discovered against Gram-positive pathogens (6). ATCC type strains were used for S. aureus, M. tuberculosis, and M. kansasii. Reference strains representative of MAC species and MAbC subspecies were selected. Growth inhibition was measured in two standard formats: in Middlebrook 7H9 growth medium using the optical density at 600 nm (OD_600_) as the readout and in cation-adjusted Mueller-Hinton broth (CAMHB) according to the CLSI guidelines (33) (Table S1). The two growth inhibition assays delivered largely similar data sets, with the exception of the MAbC strains, for which higher oxazolidinone MICs were measured in CAMHB than in Middlebrook 7H9 (Tables S2 and S3). The positive control, clarithromycin, displayed the expected patterns across all strains.

Interestingly, the potency of all the oxazolidinones was similar against the M. kansasii and M. tuberculosis strains. This constitutes a potentially important finding since sutezolid, delpazolid, and TBI-223 are in clinical development to treat TB but have not yet been considered against NTM infections caused by M. kansasii. Against the MAC and MAbC strains, the oxazolidinones were overall less potent, between 3- and >20-fold. In most cases, large shifts were observed between the MIC_50_ and MIC_90_ values, indicative of shallow dose responses. Among the MAC species, Mycobacterium chimaera was significantly and consistently more susceptible to all oxazolidinones (Tables S2 and S3). These same trends have been reported for linezolid and tedizolid against MAbC and MAC strains (5).

Because linezolid and tedizolid are approved, often used against NTM-PD, and among the most potent inhibitors of M. abscessus growth in 7H9 (Table S2), we measured their efficacy in the M. abscessus K21 NOD.CB17-Prkdc^scid^/NCrCrl (NOD SCID; Charles River Laboratories) model of acute infection (34), at the human equivalent dose (100 and 10 mg/kg for linezolid 1,200 mg and tedizolid 200 mg daily, respectively [35, 36]) and twice that dose (200 and 20 mg/kg, respectively). In this model and as single agents, neither drug caused a decrease in the M. abscessus lung burden after 10 daily doses (Fig. S2). Peak (3 h) and trough (24 h) plasma concentrations measured in 3 mice per treatment group indicated on-target exposure for linezolid and slightly above-target exposure for tedizolid (Table S4). All experiments involving live animals were approved by the Institutional Animal Care and Use Committee of the Center for Discovery and Innovation (Hackensack Meridian Health). In a different mouse model of M. abscessus ATCC 19977 infection, a statistically significant lung CFU reduction (*n* = 20 mice per group) of 0.3 log was achieved with linezolid at 100 mg/kg (37), which could be due to the difference in MIC between ATCC 19977 (MIC_50_/MIC_90_ = 0.3/1.2 μg/mL in 7H9) (Table S2) and the K21 clinical isolate used in our model (MIC_50_/MIC_90_ = 1.4/20 μg/mL in 7H9).

Next, we calculated the MPS IC_50_/MIC_50_ and IC_50_/MIC_90_ ratios or selectivity index (SI) for all oxazolidinones (Table 2), as indicators of *in vivo* safety. The same ranking of MPS/MIC ratios were observed across all oxazolidinones and species/strains whether the MICs in 7H9 or CAMHB were used (Table S5). By these metrics, TBI-223 and sutezolid consistently exhibited the most favorable SIs and comfortably met standard infectious disease guidelines of SI > 10 (where SI is the ratio between the 50% cytotoxic concentration [CC_50_] in host cells and the MIC_50_ [38], in the absence of MPS-specific SI recommendations) against M. tuberculosis and M. kansasii. While favorable, the SI of sutezolid’s active metabolite, M1, was only marginally higher than that of linezolid. Since 82% to 87% of the active drug exposure at steady state is composed of the M1 metabolite (39), this finding may reduce the superiority margin of sutezolid. Among the other oxazolidinones, contezolid exhibited the largest SI, potentially more favorable than that of linezolid for the treatment of TB. How these oxazolidinone SIs correlate with clinical toxicity will help establish the predictive value of the MPS assay in the development of better-tolerated oxazolidinone candidates.

**TABLE 2:** Comparative ratios of the MPS inhibitory concentrations to MICs or selectivity indexes as an estimate of the therapeutic window^*a*^

Drug	Drug abbreviation	MPS IC_50_ (μg/mL)	MIC_50_ in 7H9 (μg/mL) against:				Selectivity index (MPS IC_50_/MIC_50_) against:				Selectivity index (MPS IC_50_/MIC_90_) against:			
			M. tuberculosis	M. kansasii	MAC	MAbC	M. tuberculosis	M. kansasii	MAC	MAbC	M. tuberculosis	M. kansasii	MAC	MAbC
Linezolid	LZD	4.8	0.30	0.50	2.50	1.00	16	10	2	5	5	3	0.3	1.0
Tedizolid	TZD	0.2	0.10	0.20	0.75	0.25	2	1.0	0.3	0.8	0.7	0.3	0.0	0.1
Sutezolid	SZD	10	0.15	0.12	0.75	1.50	**67**	**83**	13	7	**20**	**20**	5.0	1.3
Sutezolid-M1	SZD-M1	7.8	0.40	0.75	3.00	3.00	**20**	10	3	3	8	4	0.5	0.8
Delpazolid	DZD	5.8	0.75	0.75	2.00	0.50	8	8	3	12	3	1.5	0.7	1.2
TBI-223		68	1.50	2.00	8.00	2.00	**45**	**34**	9	**34**	19	17	3.4	3.4
Radezolid	RZD	0.3	0.08	0.50	3.00	0.25	4	0.6	0.1	1.2	1.2	0.2	0.0	0.2
Contezolid	MRX-1	10.6	0.40	1.00	3.00	2.00	**27**	11	4	5	11	4	0.7	0.7

a Dark gray shading and bold typeface indicate selectivity indices of >20; light gray shading indicates selectivity indices of >10. TBI-223 and sutezolid exhibited the most attractive profiles across the species tested. The MIC values are derived from Table S2 in the supplemental material for the M. tuberculosis and M. kansasii strains; values listed in the “MAC” and “MAbC” columns represent the median MICs against 5 MAC strains and 3 MAbC strains, respectively. MPS, mitochondrial protein synthesis; MAC, M. avium complex; MAbC, M. abscessus complex.

This study presents a couple of limitations. Only a small set of mycobacterial reference strains was used to determine the activity of oxazolidinones against the various mycobacterial pathogens, and their potency was measured as single agents only. Reduced MICs against M. abscessus have been reported for linezolid combined with various drugs, including clarithromycin or amikacin (reviewed in reference 40), and more recently, for tedizolid combined with clarithromycin, potentially leading to improved therapeutic index (TI) (5).

Nevertheless, this is the first study to report side-by-side MPS inhibition data for the major oxazolidinones in clinical development and compare MPS IC_50_ values to potency data against the major pathogens responsible for TB and nontuberculous mycobacterial lung disease. We found that TBI-223 and sutezolid presented substantially higher SIs than all other oxazolidinones tested and exceeded the recommended SI, whether MIC_50_ or MIC_90_ were considered, noting the overall large shift between the two values for all oxazolidinones. We also found that several oxazolidinones were equally potent against M. kansasii and M. tuberculosis and could be considered for the treatment of NTM-PD caused by M. kansasii in addition to TB.
