# Supplementary material for: Side-by-Side Profiling of Oxazolidinones to Estimate the Therapeutic Window against Mycobacterial Infections
Source: Antimicrob Agents Chemother. 2023 Mar 15;67(4):e01655-22. doi: 10.1128/aac.01655-22 (PMC10112060; doi:10.1128/aac.01655-22)
Supplement: Supplemental file 1 — Supplemental material. Download aac.01655-22-s0001.docx, DOCX file, 2.1 MB [file aac.01655-22-s0001.docx]

Side-by-side profiling of oxazolidinones to estimate therapeutic window against mycobacterial infections

Dereje A. Negatu ^a, b^, Wassihun Wedajo Aragaw ^a^, Julianna Cangialosi ^a^, Véronique Dartois ^a, c, #^ and Thomas Dick ^a, c, d^

^a^ Center for Discovery and Innovation, Hackensack Meridian Health, Nutley, New Jersey, USA

^b^ Center for Innovative Drug Development and Therapeutic Trials for Africa (CDT-Africa), Addis Ababa University, Addis Ababa, Ethiopia

^c^ Department of Medical Sciences, Hackensack Meridian School of Medicine, Nutley, New Jersey, USA

^d^ Department of Microbiology and Immunology, Georgetown University, Washington, DC, USA

Running Title:

Keywords: Oxazolidinone, mitochondrial toxicity, tuberculosis, nontuberculous mycobacteria

^#^ Corresponding author: [veronique.dartois@hmh-cdi.org](mailto:veronique.dartois@hmh-cdi.org)

**Table S1.** Middlebrook 7H9 and CAMHB media composition, assay conditions and readouts (1)

| **Conditions** | ***S. aureus*** | **Mtb** | **Fast-growing NTMs (*M. abscessus*)** | | **Slow-growing NTMs (*M. kansasii,* MAC)** | |
| --- | --- | --- | --- | --- | --- | --- |
| Media | CAMHB ^[a]^ | Complete Middlebrook 7H9 ^[b]^ | Complete Middlebrook 7H9 | CAMHB | Complete Middlebrook 7H9 | 5% OADC CAMHB |
| pH | 7.4 | 6.8 | 6.8 | 7.4 | 6.8 | 7.4 |
| Plate format | 96-well plate | 96-well plate | 96-well plate | 96-well plate | 96-well plate | 96-well plate |
| Total volume | 100 μL | 200 μL | 200 μL | 100 μL | 200 μL | 100 μL |
| Inoculum density (OD_600_) | 0.005 | 0.05 | 0.05 | 0.005 | 0.05 | 0.005 |
| Incubation temperature | 37º | 37º | 37º | 37º | 30º | 37º |
| Shaking, rpm | standing | 90 | 90 | standing | 90 | standing |
| Length of incubation | 16 h | 7 days | 3 days | 5 days | 4 days | 10 days |
| MIC determination | Visual | OD 600 nm | OD 600 nm | Visual | OD 600 nm | Visual |

^[a]^ Composition of cation adjusted Mueller Hinton broth (CAMHB): casein acid hydrolysate, 17.5 g/L; beef extract, 3 g/L; starch, 1.5 g/L; 25 mg/L Ca^++^; 12.5 mg/L Mg^++^; 5% Middlebrook Oleic Albumin Dextrose Catalase Growth Supplement (OADC, Sigma-Aldrich) for Slow Growing NTM Species.

^[b]^ Composition of complete Middlebrook 7H9 growth medium: Ammonium Sulfate, 0.5 g/L; L-Glutamic Acid, 0.5 g/L; Sodium Citrate, 0.1 g/L; Pyridoxine, 1.0 mg/L; Biotin, 0.5 mg/L; Disodium Phosphate, 2.5 g/L; Monopotassium Phosphate, 1.0 g/L; Ferric Ammonium Citrate, 0.04 g/L; Magnesium Sulfate, 0.05 g/L; Calcium Chloride, 0.5 mg/L; Zinc Sulfate, 1.0 mg/L; Copper Sulfate, 1.0 mg; 10% Albumin Dextrose Catalase (Sigma Aldrich); 0.05% Tween 80; 0.2% Glycerol.

**Table S2.** Anti-mycobacterial activity (MIC in µg/mL) of clinically approved and advanced oxazolidinones in 7H9 medium

| Drugs / readout | | *Staphylo-coccus aureus* ATCC 12600 | *Myco-bacterium tuberculosis* H37Rv ATCC 27294 | *Myco-bacterium kansasii* Hauduroy ATCC 12478 | *Mycobacterium avium* complex | | | | | *Mycobacterium abscessus* complex | | |
| --- | --- | --- | --- | --- | --- | --- | --- | --- | --- | --- | --- | --- |
|  |  |  |  |  | *M. avium* subsp *avium* ATCC 35717 | *M. avium* subsp. *hominissuis* ATCC 700898 ^[a]^ | *M. avium* subsp *hominissuis* 109 ^[b]^ | *M. chimaera* CCUG 50989 | *M. intracellulare* ATCC 13950 | subsp *abscessus* ATCC19977 | subsp *bolletii* CCUG 50184-T | subsp *massiliense* CCUG 48898-T |
| LZD | |  |  |  |  |  |  |  |  |  |  |  |
|  | MIC_50_ | 0.25 | 0.30 | 0.5 | 3.0 | 4.0 | 2.5 | 0.75 | 0.75 | 1.0 | 1.5 | 1.0 |
|  | MIC_90_ | 1.00 | 1.00 | 1.5 | 25.0 | 16.0 | 32.0 | 3.0 | 1.5 | 3.5 | 6.0 | 5.0 |
|  | MIC_Vis_ | 2.00 | NA | 4.0 | >32.0 | >32.0 | >32.0 | 8.0 | 2.0 | 8.0 | 16.0 | 16.0 |
| TZD | |  |  |  |  |  |  |  |  |  |  |  |
|  | MIC_50_ | 0.10 | 0.10 | 0.2 | 2.0 | 1.5 | 0.75 | 0.15 | 0.25 | 0.3 | 0.25 | 0.25 |
|  | MIC_90_ | 0.25 | 0.30 | 0.75 | 6.0 | 6.0 | 8.0 | 0.75 | 0.5 | 1.5 | 3.0 | 3.0 |
|  | MIC_Vis_ | 0.50 | NA | 2.0 | 16.0 | 32.0 | 32.0 | 2.0 | 1.0 | 2.0 | 8.0 | 8.0 |
| SZD | |  |  |  |  |  |  |  |  |  |  |  |
|  | MIC_50_ | 0.50 | 0.15 | 0.12 | 0.75 | 1.0 | 0.75 | 0.3 | 0.3 | 1.5 | 1.5 | 1.5 |
|  | MIC_90_ | 1.50 | 0.50 | 0.5 | 2.0 | 3.0 | 4.0 | 0.75 | 0.4 | 5.0 | 8.0 | 8.0 |
|  | MIC_Vis_ | 2.00 | NA | 2.0 | 4.0 | 16.0 | 16.0 | 1.0 | 0.5 | 8.0 | 16.0 | 16.0 |
| SZD-M1 | |  |  |  |  |  |  |  |  |  |  |  |
|  | MIC_50_ | 3.00 | 0.40 | 0.75 | 4.0 | 4.0 | 3.0 | 0.75 | 0.25 | 2.0 | 3.0 | 4.0 |
|  | MIC_90_ | 6.00 | 1.00 | 2.0 | 32.0 | 32.0 | >32.0 | 2.0 | 0.75 | 8.0 | 10.0 | 12.0 |
|  | MIC_Vis_ | 8.00 | NA | 4.0 | >32.0 | >32.0 | >32.0 | 4.0 | 1.0 | 16.0 | 32.0 | >32.0 |
| DZD | |  |  |  |  |  |  |  |  |  |  |  |
|  | MIC_50_ | 0.75 | 0.75 | 0.75 | 3.0 | 3.0 | 2.0 | 0.75 | 0.75 | 0.5 | 0.5 | 0.75 |
|  | MIC_90_ | 1.50 | 2.00 | 4.0 | 12.0 | 8.0 | 16.0 | 2.0 | 1.5 | 3.5 | 5.0 | 5.0 |
|  | MIC_Vis_ | 2.00 | NA | 8.0 | 32.0 | 32.0 | >32.0 | 4.0 | 2.0 | 4.0 | 8.0 | 8.0 |
| TBI-223 | |  |  |  |  |  |  |  |  |  |  |  |
|  | MIC_50_ | 3.00 | 1.50 | 2.0 | 12.0 | 12.0 | 8.0 | 3.0 | 2.0 | 1.5 | 2.0 | 4.0 |
|  | MIC_90_ | 4.00 | 3.50 | 4.0 | 32.0 | 32.0 | >32.0 | 8.0 | 3.0 | 10.0 | 20.0 | 20.0 |
|  | MIC_Vis_ | 8.00 | NA | 8.0 | >32.0 | >32.0 | >32.0 | 16.0 | 8.0 | 16.0 | 32.0 | >32.0 |
| RZD | |  |  |  |  |  |  |  |  |  |  |  |
|  | MIC_50_ | 0.60 | 0.08 | 0.5 | 3.0 | 4.0 | 3.0 | 0.75 | 0.13 | 0.2 | 0.25 | 0.25 |
|  | MIC_90_ | 1.50 | 0.25 | 2.0 | 30.0 | 32.0 | >32.0 | 1.5 | 0.25 | 1.5 | 1.5 | 3.0 |
|  | MIC_Vis_ | 2.00 | NA | 4.0 | >32.0 | >32.0 | >32.0 | 4.0 | 1.0 | 2.0 | 4.0 | 4.0 |
| CZD | |  |  |  |  |  |  |  |  |  |  |  |
|  | MIC_50_ | 0.06 | 0.80 | 1.5 | 2.0 | 1.5 | 1.5 | 0.75 | 0.25 | 1.5 | 2.0 | 4.0 |
|  | MIC_90_ | 1.50 | 1.50 | 16.0 | 32.0 | 4.0 | 4.0 | 2.0 | 0.75 | 8.0 | >32.0 | >32.0 |
|  | MIC_Vis_ | 2.00 | NA | >32.0 | >32.0 | >32.0 | >32.0 | 4.0 | 1.0 | >32.0 | >32.0 | >32.0 |
| MRX-1 | |  |  |  |  |  |  |  |  |  |  |  |
|  | MIC_50_ | 0.75 | 0.40 | 1.0 | 4.0 | 4.0 | 3.0 | 0.75 | 3.0 | 1.0 | 2.0 | 3.0 |
|  | MIC_90_ | 1.50 | 1.00 | 3.0 | 20.0 | 16.0 | 32.0 | 3.0 | 6.0 | 8.0 | 20.0 | 16.0 |
|  | MIC_Vis_ | 2.00 | NA | 8.0 | 32.0 | >32.0 | >32.0 | 8.0 | 8.0 | 16.0 | 32.0 | 32.0 |
| CLR | |  |  |  |  |  |  |  |  |  |  |  |
|  | MIC_50_ | ND | ND | 0.13 | 0.25 | 0.4 | 0.75 | 0.1 | 0.5 | 0.1 | 0.25 | 0.05 |
|  | MIC_90_ | ND | ND | 0.5 | 2.0 | 1.0 | 4.0 | 0.25 | 1.5 | 2.0 | 2.0 | 0.2 |
|  | MIC_Vis_ | ND | ND | 2.0 | 4.0 | 8.0 | 16.0 | 0.5 | 16.0 | 4.0 | 4.0 | 0.5 |

^[a]^ ATCC 700898 also known as Chester, *Mycobacterium avium* subsp *hominissuis* 101 (2)

^[b]^ whole genome sequenced (3)

MIC_50_ and MIC_90_: lowest concentrations that inhibit 50% and 90% of growth, respectively. MIC_Vis_: lowest concentration that suppress visible growth or presence of turbidity as assessed by visual inspection.

**Table S3.** Anti-NTM activity (MIC in µg/mL) of clinically approved and advanced oxazolidinones in CAMHB

| Drugs / readout | | *Mycobacterium kansasii* Hauduroy ATCC 12478 | *Mycobacterium avium* complex | | | | | *Mycobacterium abscessus* complex | | |
| --- | --- | --- | --- | --- | --- | --- | --- | --- | --- | --- |
|  |  |  | *M. avium* subsp *avium* ATCC 35717 | *M. avium* subsp. *hominissuis* ATCC 700898^[a]^ | *M. avium* subsp *hominissuis* 109 ^[b]^ | *M. chimaera* CCUG 50989 | *M. intracellulare* ATCC 13950 | subsp *abscessus* ATCC19977 | subsp *bolletii* CCUG 50184-T | subsp *massiliense* CCUG 48898-T |
| LZD | |  |  |  |  |  |  |  |  |  |
|  | MIC_50_ | 0.8 | 4 | 4 | 2.5 | 1.5 | 1 | 12 | 7 | 7 |
|  | MIC_90_ | 1.5 | 16 | 32 | 25 | 8 | 2 | >32 | 32 | 25 |
|  | MIC_Vis_ | 4 | >32 | >32 | >32 | 16 | 4 | >32 | >32 | 32 |
| TZD | |  |  |  |  |  |  |  |  |  |
|  | MIC_50_ | 0.4 | 1 | 1 | 1 | 0.4 | 0.4 | 2 | 4 | 1 |
|  | MIC_90_ | 0.8 | 5 | 8 | 10 | 1.5 | 0.8 | 6 | 8 | 3 |
|  | MIC_Vis_ | 2 | 16 | >16 | 16 | 4 | 1 | 8 | 16 | 8 |
| SZD | |  |  |  |  |  |  |  |  |  |
|  | MIC_50_ | 0.2 | 0.5 | 1.5 | 1.5 | 0.5 | 0.4 | 10 | 12 | 2 |
|  | MIC_90_ | 0.25 | 3 | 4 | 4 | 1 | 0.8 | 20 | 25 | 10 |
|  | MIC_Vis_ | 0.5 | 8 | 8 | 8 | 2 | 1 | >32 | >32 | 16 |
| SZD-M1 | |  |  |  |  |  |  |  |  |  |
|  | MIC_50_ | 1 | 2 | 4 | 3 | 4 | 0.8 | 20 | 18 | 7 |
|  | MIC_90_ | 3 | 16 | >32 | >32 | 16 | 1.2 | >32 | >32 | >32 |
|  | MIC_Vis_ | 8 | >32 | >32 | >32 | >32 | 2 | >32 | >32 | >32 |
| DZD | |  |  |  |  |  |  |  |  |  |
|  | MIC_50_ | 1.5 | 2 | 5 | 4 | 1 | 1.2 | 7 | 4 | 2 |
|  | MIC_90_ | 2.5 | 16 | 16 | 16 | 3 | 2.4 | 16 | 16 | 10 |
|  | MIC_Vis_ | 4 | >32 | >32 | >32 | 8 | 4 | 32 | 32 | 16 |
| TBI-223 | |  |  |  |  |  |  |  |  |  |
|  | MIC_50_ | 1 | 6 | 9 | 12 | 4 | 2.4 | 27 | 25 | 16 |
|  | MIC_90_ | 2 | 32 | 32 | >32 | 32 | 4.6 | >32 | >32 | >32 |
|  | MIC_Vis_ | 4 | >32 | >32 | >32 | >32 | 8 | >32 | >32 | >32 |
| RZD | |  |  |  |  |  |  |  |  |  |
|  | MIC_50_ | 0.5 | 2 | 8 | 2 | 1 | 0.4 | 4 | 3 | 1.5 |
|  | MIC_90_ | 2 | 16 | >32 | >32 | 2 | 0.6 | 12 | 10 | 5 |
|  | MIC_Vis_ | 4 | >32 | >32 | >32 | 8 | 1 | 32 | 32 | 8 |
| CZD | |  |  |  |  |  |  |  |  |  |
|  | MIC_50_ | 3 | 1 | 1 | 1 | 1.5 | 0.6 | >32 | >32 | >32 |
|  | MIC_90_ | 16 | 25 | 32 | >32 | 32 | 1.2 | >32 | >32 | >32 |
|  | MIC_Vis_ | >32 | >32 | >32 | >32 | >32 | 2 | >32 | >32 | >32 |
| MRX-1 | |  |  |  |  |  |  |  |  |  |
|  | MIC_50_ | 0.1 | 2 | 3 | 4 | 3 | 4 | >32 | >32 | >32 |
|  | MIC_90_ | 2 | >32 | 32 | >32 | 10 | 8 | >32 | >32 | >32 |
|  | MIC_Vis_ | 4 | >32 | >32 | >32 | 16 | 16 | >32 | >32 | >32 |
| CLR | |  |  |  |  |  |  |  |  |  |
|  | MIC_50_ | 0.05 | 0.1 | 0.4 | 0.2 | 0.1 | 2 | 0.2 | 0.2 | 0.05 |
|  | MIC_90_ | 0.2 | 1 | 1 | 3 | 0.8 | 3 | 3 | 3 | 0.2 |
|  | MIC_Vis_ | 0.5 | 4 | 2 | 4 | 1.5 | 4 | 4 | 4 | 0.25 |
| CLR ^[a]^ | |  |  |  |  |  |  |  |  |  |
|  | MIC_50_ | NA | NA | NA | NA | NA | NA | >16 | 16 | 0.03 |
|  | MIC_90_ | NA | NA | NA | NA | NA | NA | >16 | >16 | 0.25 |
|  | MIC_Vis_ | NA | NA | NA | NA | NA | NA | >16 | >16 | 0.5 |

^[a]^ CLR MIC determined after 14 days of incubation against *M. abscessus* subsp *abscessus* ATCC19977 and *M. abscessus* subsp *bolletii* CCUG 50184-T which harbor a functional *erm*41 (ribosome methylase) allele conferring resistance to CLR, and against *M. abscessus* subsp *massiliense* which harbors an inactive *erm*41 allele (4)

MIC_50_ and MIC_90_: lowest concentrations that inhibit 50% and 90% of growth, respectively. MIC_Vis_: lowest concentration that suppresses visible growth or presence of turbidity as assessed by visual inspection.

**Table S4**. Plasma concentrations of linezolid and tedizolid in infected SCID mice on treatment, at peak and trough

|  | Linezolid 100 mg/kg | | Linezolid 200 mg/kg | | Tedizolid 10 mg/kg | | Tedizolid 100 mg/kg | |
| --- | --- | --- | --- | --- | --- | --- | --- | --- |
|  | Peak (3h) | Trough (24h) | Peak (3h) | Trough (24h) | Peak (3h) | Trough (24h) | Peak (3h) | Trough (24h) |
| Mouse 1 | 29,100 | 49 | 40,500 | 265 | 5,220 | n.d. | 23,700 | 579 |
| Mouse 2 | 21,800 | n.d. | 89,700 | 70 | 7,170 | 449 | 10,100 | 4,870 |
| Mouse 3 | 34,400 | 33 | 81,800 | 74 | 4,630 | 656 | 22,800 | 4,330 |
| Average (ng/mL) | 28,433 | 41 | 70,667 | 136 | 5,673 | 553 | 18,867 | 3,260 |
| SD (ng/mL) | 6,326 | 12 | 26,422 | 111 | 1,329 | 146 | 7,605 | 2,337 |
| AUC estimate ^[a]^ (μg*h/mL) | 342 (76) | | 850 (316) | | 75 (15) | | 266 (76) | |
| Clinical AUC (μg*h/mL) | 274 (range 148–721) at 1200 mg once daily (5) | | n.a. | | 6.6 to 49.9 at 200 mg once daily (6) | | n.a. | |

^[a]^ Area under the curve, average (standard deviation, n=3), only broad estimates are provided due to the limited peak and trough sampling

**Table S5**. Comparative ratios of MPS inhibitory concentrations to MIC measured in cation adjusted Mueller Hinton broth (CAMHB)

**Figure S1. Mitochondrial protein synthesis inhibition assays using the MitoBiogenesis™ In-Cell ELISA Kit** (ab110217, Abcam). HepG2 cells (ATCC HB-8065) were grown at 37°C, 5% CO_2_, 90% relative humidity to 60% confluence in T75 flasks in a growth medium consisting of DMEM (Corning#10-013-CV) containing 4.5 g/L glucose and 4 mM L-glutamine supplemented with 10% FBS and 1mM sodium pyruvate. Cells were trypsinized in 0.05% trypsin-EDTA (Gibco) and re-suspended in 10 mL growth medium. Cells were filtered through a 40 µm filter and seeded in collagen-coated 96-well plates (Gibco#A1142803) at 6,000 cells/well in a volume of 200 μL/well with plates agitated upon cell addition to ensure even distribution. After overnight incubation, media were aspirated from the 96-well plate and replaced with 200 μL of growth medium with serially diluted compound ensuring a duplicate 10-point twofold titration series, ensuring that the final DMSO concentration did not exceed 0.5% with duplicate drug-free vehicle control wells as well as drug-free controls in duplicate for the background as detailed below. Chloramphenicol and clarithromycin were used as positive and negative controls. To prepare stock solution, all oxazolidinones and clarithromycin were dissolved in DMSO at 10 mM, except TBI-223 which was dissolved at 80 mM to test concentrations up to 128 ug/mL in the MPS assay. Chloramphenicol was dissolved in ethanol at 100 mM. Each well was inspected under an inverted microscope to detect compound precipitation, which was not observed in this study. The plates were incubated an additional 72 h after which the medium was gently aspirated and replaced with 4% paraformaldehyde (100 μL/well) (Fisher Scientific#50-980-493). After 20 min fixation, the medium was removed, and wells washed three times with 200 μL/well PBS. Wells were treated for 5 min with 0.5% acetic acid (100 μL/well), wells were washed with 200 μL/ well PBS and subsequently treated with permeabilization buffer (0.1 % Triton X-100) (100 μL/well) for 30 min. Buffer was removed and replaced with 2× blocking buffer from the MitoBiogenesis™ In-Cell ELISA Kit (ab110217, Abcam). After 2 h incubation, primary antibody was added (100 μl/well) in 1× incubation buffer per manufacturer’s recommendation except for the background control wells which received 100 μl/well 1× incubation buffer only. Plates were incubated overnight at 4 °C after which medium was removed and wells washed three times at 200 μl/well with wash buffer (0.25% Tween-20 in PBS). Alkaline phosphatase/ horse radish peroxidase-labeled secondary antibody solution prepared according to the manufacturer’s instructions in 1× incubation buffer was added at 100 μl/well to all wells. After 1 h incubation, wells were washed 4 times with wash buffer (200 μl/well) followed by addition of 100 μl/well freshly prepared per manufacturer’s recommendation alkaline peroxidase development solution. The reaction was monitored at 405 nm in a TECAN Infinite Pro 200 plate reader. The reagent was removed, and 100 μl/well horse radish peroxidase development solution added. The reaction was monitored at 600 nm in the microplate reader after 15 min incubation. The development solution was completely removed, and plates blotted to dryness after which 1× Janus Green stain (50 μl/well) was added. Plates were washed 5 times with water after 5 min, 0.5 M HCl (100 μl/well) added, incubated for 10 min with shaking and absorbance recorded at 595 nm. Data was interpreted per manufacturer’s instructions and plotted in GraphPad Prism to calculate IC_50_ values for COX-1/SDHA and cell viability.

**Figure S2.** Efficacy of linezolid and tedizolid in the SCID mouse model of acute *M. abscessus* infection. Eight-week old female NOD SCID mice were infected intranasally with 10^6^ CFU of *M. abscessus* K21 as described previously (7) (7). The MIC_50_ and MIC_90_ of linezolid against M. abscessus K21 are 1.35 and 20 μg/mL, respectively. The MIC_50_ and MIC_90_ of tedizolid against M. abscessus K21 are 0.15 and 4.6 μg/mL, respectively. Linezolid (LZD) and tedizolid (TZD) were administered orally once daily for six days at 100 (LZD) and 200 (hiLZD) mg/kg, and 10 (TZD) and 20 (hiTZD) mg/kg, starting one day post-infection. These doses were selected to reproduce the exposure achieved at the clinically used dose in patients (LZD and TZD arms) and twice that dose (hiLZD and hiTZD arms). All mice were euthanized 24h after the last dose. Bacterial loads were quantified by plating serial dilutions of lung homogenates on Middlebrook 7H11 agar. Treatment with vehicle alone did not affect the bacterial lung burden (D11).

REFERENCES

1. CLSI. 2018. Susceptibility testing of Mycobacteria, Nocardia spp., and Other Aerobic Actinomycetes. Clinical and Laboratory Standards Institute, Wayne, PA.

2. Andrejak C, Almeida DV, Tyagi S, Converse PJ, Ammerman NC, Grosset JH. 2015. Characterization of mouse models of Mycobacterium avium complex infection and evaluation of drug combinations. Antimicrob Agents Chemother 59:2129-35.

3. Matern WM, Bader JS, Karakousis PC. 2018. Genome analysis of Mycobacterium avium subspecies hominissuis strain 109. Sci Data 5:180277.

4. Brown-Elliott BA, Vasireddy S, Vasireddy R, Iakhiaeva E, Howard ST, Nash K, Parodi N, Strong A, Gee M, Smith T, Wallace RJ, Jr. 2015. Utility of sequencing the erm(41) gene in isolates of Mycobacterium abscessus subsp. abscessus with low and intermediate clarithromycin MICs. J Clin Microbiol 53:1211-5.

5. Imperial MZ, Nedelman JR, Conradie F, Savic RM. 2022. Proposed Linezolid Dosing Strategies to Minimize Adverse Events for Treatment of Extensively Drug-Resistant Tuberculosis. Clin Infect Dis 74:1736-1747.

6. Flanagan S, Passarell J, Lu Q, Fiedler-Kelly J, Ludwig E, Prokocimer P. 2014. Tedizolid population pharmacokinetics, exposure response, and target attainment. Antimicrob Agents Chemother 58:6462-70.

7. Dick T, Shin SJ, Koh WJ, Dartois V, Gengenbacher M. 2020. Rifabutin Is Active against Mycobacterium abscessus in Mice. Antimicrob Agents Chemother 64.
